# World Risk Society and Constructing Cosmopolitan Realities: A Bourdieusian Critique of Risk Society

**DOI:** 10.3389/fsoc.2022.797321

**Published:** 2022-04-29

**Authors:** Abbas Jong

**Affiliations:** Department of Social Sciences, Humboldt University of Berlin, Berlin, Germany

**Keywords:** Ulrich Beck, risk society, individualization, reflexive modernity, cosmopolitanism, methodological cosmopolitanism, Pierre Bourdieu, cosmopolitan field

## Abstract

Current global crises and threats have revealed the growing implications of Ulrich Beck's theory of risk society. Rather than being a theory of risk, risk society theory is more a social theory of the new social world and modernity. Risk society theory encompasses a new social ontology of the social in the era of uncertainties and crises. Beck also proposes the cosmopolitan outlook and particularly methodological cosmopolitanism as the epistemology and methodology of the world risk society. Yet, a close examination of Beck's social theory reveals a contradiction between the two aspects. On the one hand, in the ontological dimension, we are faced with the primacy of the indeterminate and the empirical, but on the other hand, Beck's epistemological prescriptive eliminate the possibility of reaching them. The current article aims to address this incompatibility. In doing so, first, the main pillars of risk society theory, and then the cosmopolitan outlook and sociology are discussed. By criticizing Beck's epistemological apparatus as well as juxtaposing the theory of risk society and Pierre Bourdieu's theory of action and fields, in the final section, the article proposes a solution to complete the ontology of risk society and overcome some of its epistemological problems.

## Introduction

In the mind of many social scientists, the name of Ulrich Beck is primarily associated with the idea of *Risk Society*. The publication of his outstanding work attracted a great deal of attention precisely in the midst of the Chernobyl nuclear crisis. In *Risk Society* [1992 (1986)], by addressing the increasing role of risk, Beck intended to reflect on a kind of shift from the first modernity to the second modernity. In the risk society, first of all, the main conflict is constituted over the distribution of bads (risks and threats) instead of the circulation of goods (capital and wealth); Secondly, in the path of greater freedom and independence for modern actors, through this transition, a kind of individualization has emerged, an individualization that has now placed the individual at the center of the structure of second modernity and provided the ground for the domination of a kind of uncertainty and ambivalence; Thirdly, de-standardization of labor in this society has led to a sharp increase in career life instability and has left people under radical uncertainty. He argues that modernization in the first phase was accompanied by the generation of issues and side effects, the effects that led to the formation of new modernity in which uncertainty and fluidity along with a kind of self-reflection—for institutions and individuals—become the prominent traits. From an epistemological point of view, Beck claims that understanding second modernity and its subsequent cosmopolitanism requires a kind of transition from methodological nationalism to methodological cosmopolitanism, a turn that is crucial in the social sciences for understanding new and really existing global phenomena that transcend old, unreal nation-state boundaries.

Due to the growing importance and influence of crises and risks in the contemporary world, many researchers attempt to study the theory of risk society to understand quiddity and implications of emerging risks, to compare different theories about risk or to critique it as a general theory of risk (O'Malley, 2009; Krahmann, 2011; Gross, 2016; Sørensen, 2018). But a closer look at Beck's works and his intellectual career would reveal that, indeed, he was not a theorist of risk, but a social theorist who attempts to introduce, understand and explain a new world. The novelty of Beck's theory of risk will be precisely understood in relation to his general social theory. Therefore, as only one part of Beck's general social theory, the theory of risk society will be effectively conceivable only in relation to the other notions as well as the methodology proposed by him (Bronner, 1995; Selchow, 2016; Burgess et al., 2018). His notions and works around individualization, uncertainty, globalization, reflexive modernity, cosmopolitanism, etc. are all components of the puzzle that make up Ulrich Beck's social theory. Beck's social theory, like other social theories, contains ontological premises of the social and their epistemological requirements. As Beck argues, recognizing the new realities of the world requires a new conceptualizing apparatus, one that can make sense of the instability, transnationality, and multifaceted nature of emerging social realities (Mythen et al., 2018). In his early works, cosmopolitan sociology and methodological cosmopolitanism are the proposing theoretical devices to take into account the process of cosmopolitanization of realities. Examining Beck's ontological premises and promises in relation to their associated epistemological propositions shows a kind of incompatibility. Beck has not proposed “a precise sketched guideline as well as a comprehensive methodological toolbox” for how the concrete and empirical consideration of his new world must be observed (Selchow, 2016). Moreover, in terms of empirical research agendas, he does not specify what the implications and requirements of methodological cosmopolitanism exactly entail. While he highlights a kind of gap between the conventional social sciences and the really existing realities, and although he prioritizes empirical categories and their empirical analysis over theoretical ones, his conceptual apparatus is, on the one hand, neutral and passive in touching the empirical realities and is not able to understand their differences, and on the other hand, a priori, he strives to impose some propositions and general categories on the empirical realities. Put differently, while his ontology in the risk society provides the ground for a kind of ontology of the contingent, by determining the contingent a prior, its epistemological propositions eliminate the possibility of reaching these contingent realities.

The main aim of the current article is to identify and critique this incompatibility and propose a solution to overcome this problem. If we consider Ulrich Beck's social ontology as an incomplete ontology of the indeterminate and the contingent, then, by criticizing his epistemological apparatus, we can complete this ontology. Given the similarities between premises and promises of Beck's and Pierre Bourdieu's social theory, this article argues that the social ontology of Beck' social theory could be completed by juxtaposing the two theories. It will be attempted to overcome some of the epistemological problems posed in cosmopolitan sociology by incorporating the theory of risk society as the unit of reference for Bourdieu's theory of action and fields. First, the theory of risk society will be introduced as a new theory of modernity—a new ontology of the novel world—and its three main dynamisms as well as two important outcomes will be examined. In the second section, the epistemology and methodology of the risk society, i.e., the cosmopolitan outlook and sociology, and their main pillars will be elaborated. Finally, by criticizing Beck's proposed epistemology and conceptualization apparatus for the global risk society, this article will address the outcomes and implications of incorporating the global risk theory as the unit of reference for Bourdieu's theories.

## Risk Society: Putting Step into the New Modernity

According to Ulrich Beck, modernity is conceivable in two phases: first, classical modernization which came to exist by breaking with traditional norms and values and generated a new industrial society from the traditional and feudal (pre-modern) society; and then, from the middle of the twentieth century (Post-WWII), these industrial societies experienced a new modernization, a modernization that radicalized and heightened modernity and its features and provided the ground for the emergence of risk society (Beck, 1992, p. 20). The principles of rationality, scientific prediction, and control are meant to lead modern industrial society, but what has transpired is that irrational strategies and policies have produced an ecological, structural, and cultural crisis. Therefore, risk society has emerged as a result of industrial society. The over/mis-consuming of natural resources, on which the first modernity was founded, has resulted in an ecological crisis (Beck, 1994). While, in the first modernity were the hazards and dangers arising from nature and over human control—such as earthquakes, floods and famine -, the hazards that modern man was able to control through the industrialization and growth of modern science, in the second modernity the threats—such as global financial crisis, terrorism, global warming, air pollution and nuclear accidents—emerging from collective human actions as well as political and economic policies in industrial societies as a result of their efforts to control the threats as well as making new opportunities. Beck has often mentions that in the second modernity, the heavy dependence on modern science and technology, along with institutions such as the state and the market in order to control the existing risks, are themselves the most important sources in creating unpredictable and unmanageable consequences in the contemporary world (Beck, 1992, 1999b).

Here, the origin and nature of change have transformed and the efforts of different societies to control and regulate nature as well as themselves have become another source for novel changes. In short, while modernization reduces risk in some social realms and forms of life, it also produces new risks that were generally previously unknown (Beck, 1992, 1999b). Institutions intended to exert control, according to Beck, manufactured uncertainty and uncontrollability, resulting in a risk society as an outcome of the “organized irresponsibility” of the modern, national and rational society (Beck, 2007a, p. 27). Because of the globality as well as unpredictability of the “mega-hazards” in the risk society, rationality and other assumed foundations of the first modernization's institutions have been dismantled. The risks escape from conventional institutional practices and policies of regulation and control. Within the risk society, threats ascend as unintended “side effects” of scientific and technological and economic progress (Mythen and Walklate, 2016). In this fashion, the production of “manufactured risks” through industrial society and scientific progress essentially makes the second modernity “a problem for itself” (Beck, 1997).

In the book of risk society, Ulrich Beck points out three central forces in second modernity, forces that have grown in company with classical modernization but have acquired considerable intensity and decisiveness in the risk Society. In the risk society, instead of distributing the goods (capital and wealth), we are undergoing the distribution of the bads (risks and threats), a new allocation that has diverse consequences for different societies; a distinctive individualization prevails in this society; and finally, work and occupations are widely de-standardized. In risk society, the main concern of individuals has shifted from natural disasters to the dangers of human activities, which are often global (Beck, 1992). According to Beck, if the driving force of class and industrial society was summed up in the slogan that “I am hungry”, the slogan of the new society is: “I am afraid” (Beck, 1992). In the former class society, the main challenge was to have a portion of society's income and benefits, and equality or inequality, but the central idea of our society is safety, and in the meantime, everyone tries to stay secure from risks and unforeseen threats. Beck argues that while pre-modern societies were cohesion based on need, solidity in the contemporary world is established based on anxiety. This means that societies were organized in a way to protect themselves from scarcity (Beck, 1992, p. 51–2; Beck, 1999b). Although contemporary societies are often societies of abundance and profusion, they are tied to each other by the new nature and increasing determinability of risks on a global scale, the risks that modern states aim to limit and regulate (Beck, 1992, p. 26). In post-scarcity society, wealth production continues to be accompanied by risk production, some of which are threatening to different societies on a global scale. In the society, then, the main conflict is not just about the distribution of wealth and capital, but also about the bads, which are various unanticipated and indeterminate types of risk, the threats that move easily between social classes, national borders and even generations (Beck, 1992, p. 19–26).

Intensified individualization is another key force of the second modernity. In the second modernity, with the decline of the authority of the dominant institutions alongside the deteriorating determinants of social classes as overriding social entities, it is the individuals who have to face the threats and find a way to reduce the effects of risks on their lives (Beck, 1992, p. 87–90). Individualization entails the destruction of industrial society's certainties, as well as the need to develop new certainties for individuals when they can no longer rely on previous ones. Here we are confronted with a vast global entanglement between individuals. Hence, individualization and globalization are two interdependent and driving forces of the second modernity (Beck et al., 2003). According to Beck, the individual has become dis-embedded from social institutions in the second modernity without being re-embedded (Rossi, 2014, p. 61). As a result, individualism is no longer routinized but rather radicalized, and this “dis-embedded individualization becomes the driving force of the second modernity” (Beck and Williams, 2004, p. 63; Rossi, 2014).

Opportunities, threats, and ambivalences in human life that were previously possible to overcome in the family, community, or by belonging to a group or social class must be perceived, interpreted, and controlled by individuals themselves (Beck, 1994, 1997). Institutions and structures are no longer the sources of individuals' life, but it is the individual who can be the source of the meaning of life as well as certainty, and consequently, the legitimacy and prominence of institutions are questioned. The restriction of religion to the private sphere, the expansion of nuclear families, and the emergence of welfare states are all symbols of this transformation (Beck, 1999a, 2007a, p. 54–55). As Beck put it, this process leads to the emergence of an “individualized society”, a process that encompasses all aspects of Individual's lives from lifestyle to institutions, their existence and relationships, and subsequently itself gives rise to a new subjectivity (Beck, 1992).

In the light of this process, according to Beck, at the end of the twentieth century, individuality has intensified and the modern world has moved from structure to agency. Life in the second modernity is characterized by the power of choice for individuals, the capability that was unknown for previous generations. Beck calls this process the “institutionalization of individualism,” a process that, while it may bring new freedoms to the individual, imposes new responsibilities on them, a new burden that has arisen as a result of declining the authority of prominent institutions (Beck and Beck-Gernsheim, 2002). We have now entered a world of uncertainty and anxiety. When it comes to making decisions, individuals are free and alone, but they are still alone when it comes to dealing with the consequences of their decisions. The transformations in the nature of social risks and threats as well as the institutionalization of individualism, drive a basic shift in the nature of politics and in patterns of social configuration and then prioritizes the empirical politics of life over other aspects of social life (Beck, 1992, 1994, 1997, 1999b; Beck and Beck-Gernsheim, 2002). The simultaneous processes of risk generation and individualization have become the driving forces of social change. Hence, the modern subject cannot be indifferent to these transformations in society. The large-scale production of manufactured risks has negative repercussions in a variety of social realms, forcing people to re-think their lives as planned projects and becoming individually accustomed to dealing with risk and uncertainty (Mythen and Walklate, 2016, p. 405).

The process of extending risks coupled with the collapse of the certainties of the industrial society which causes a profound change in the form and content of work. In respect to the life careers, if in industrial society labor and work were linear and standardization, in the risk society it would take on a non-linear and unpredictable state, and become de-standardization. If in the industrial society individuals' job determined what their system of life should be like, today the factors that shape the order and system of individuals' life have diversified. The emergence of new forms of employment and unemployment through work at home or work in cyberspace has also changed the form of employment contracts. In some aspects, this generates risks, and he claims that it leads to pluralized underemployment, which correspondingly makes it difficult to rely on former social protection systems. The dominance of uncertainty over work and the impact of national and transnational factors on it will have serious consequences for social organizations as well as individual life (Beck, 1992, p. 141–150; Beck, 2007a). Therefore, in the risk society full-time employment and long-term careers are no longer possible, and people's status and patterns of consumption as well as identity are no longer determined by their socio-economic positions (Rossi, 2014; Jong, 2016a,b). Individuals find themselves alone in interconnected but highly insecure and fluid social relations, and they have to solve their social problems and plan their lives on their own.

Beck averred that it is not only the biological threats, and health or medical risks that dominate risk society, but that society also encompasses a vast network of interrelated changes in social life; Changes in employment patterns, distrust of reference institutions such as science and state, reduction of the penetration and authority of traditions and customs on individual identity, the disintegration of family patterns, the democratization of personal relationships, unbridled developments, etc. (Beck, 1992, 1999b, 2007b). In this fluid and uncertain world, all kinds of decisions will bring risks to individuals. According to Beck, these changes are not a symbol of going beyond modernity but of its heightening. Beck argues that while modernization will rise a large number of challenges and contradictions that affect modern institutions, the weight and complexity of the issues will drive the institution into a self-evaluation process, forcing them to develop a new form of self-reflection (Beck, 1992). Hence, from inside of the industrial society, we are witnessing the emergence of a new modernization, which he calls reflexive modernization, a modernization that is accompanied by a heightened awareness of human non-knowledge and his or her inability to dominate nature, technique, society, and so on (Beck, 1992; Beck et al., 2003). According to him, risk society and individualization change the foundations and institutions of the first modernity and provide the ground for the development of reflexive modernization (Beck et al., 2003). Thus, although modernization has shaped an industrial society and a risk society, which is surrendered by threats and crises caused by non-human rational factors, it has also made it possible for a human to have self-reflexing about themselves as well as and their social environment, a self-reflection which reconstruct their subjectivity and individuality together with and their community. Beck argue that at the core of reflexive modernity lies a dual dynamism: Simultaneous process of inventive self-deconstruction, or the dis-embedding of industrial social forms and formations—such as class, stratum, occupation, gender, family, and economy—; and the process of re-construction, or the re-embedding of society with new but unknown social formations (Beck, 1997).

The first step of reflexive modernization includes a self-encounter with the impacts of risk society that are incompatible with the industrial society and its relevant institutionalized standards (Beck, 1994). Since risks and threats are beyond human awareness, imagination, and scientific determination, in a risk society, industrial dangers become more prevalent, but no standards of responsibility or monitoring of the threats can be established. As a result of this ongoing process, the second dynamism of reflexive modernization gets under way, a process which include the re-construction or the re-modernization of society with a new form of subjectivity, individuality, family, capitalism, state, labor, globalization, everyday life, etc. (Beck, 1994; Beck et al., 2003; Rossi, 2014).

In this respect, Beck distinguishes between the “reflective individual” of the first modernity and the “reflexive individual” as the main actor of the second modernity and risk society. The duality of the subject and object, placing the object in front of a rational subject, and the availability of certain and objective knowledge are all the main pillars of reflection. In second modernity and under the all-encompassing penetration of risks, the individual is compelled to make numerous and quick decisions without following pre-existing instructions. This circumstance causes the individual to function as a reflex or an incessant manufacturer of uncertain and instantaneous reflexes: agreements, networks, and unifications are constantly constructed, de-constructed, and re-constructed (Beck, 1992; Beck et al., 2003). Consequently, an individualization of self-definitions, self-consciously constructed categories and boundaries comes into being (Rossi, 2014). Confronting these categories and boundaries as well as institutional and individual troubles form the nature of the individualization. In this circumstance, individuals are both the creator of these categories, boundaries and troubles, and also their outcomes. Society and the individual are constructed and re-constructed in tandem through self-choosing, self-defining and self-organizing activities. As a result, the hallmarks of reflexive modernity include ambivalence, contradiction, and internalization of indeterminacy (Beck et al., 2003, p. 21–25; Rossi, 2014).

Beck argue that threats and risks in a risk society have no spatial, temporal or social constraints and affect all societies and social classes. The global outcomes of the ongoing threats transcend national borders and turn the risk society into a society at global risk (Beck, 2007b) or in Beck's words, a world risk society (Beck, 1999b). Therefore, facing these threats and challenges requires a global, transnational outlook. On this basis, he believes that the most important aspect of risk society is cosmopolitanization, which means internal globalization from within national societies. Cosmopolitanization significantly transforms everyday consciousness and identities (Beck, 1999a,b, 2006, 2007b). Global challenges and worries are becoming part of the local everyday experience and moral life of people. Global risks have formed a comprehensive vision for humanity. According to Beck, if the first modernity was national modernity, the second modernity is transnational modernity or cosmopolitan modernity. Second modernity comes about when society ceases to be equivalent to the nation-state when socio-economic, cultural, political, and technological development becomes fundamentally transnational (Beck, 2016d). In cosmopolitanism, national structures and organizations are losing their significance at a high level, and we are experiencing the cosmopolitanization of nation-states from within (Beck, 2016c). Cosmopolitanization as an ontological transition also entails special epistemological and methodological implications in the social sciences. He considered cosmopolitan outlook and methodological cosmopolitanism as the key epistemological framework and methodological toolbox for understanding the risk society and the cosmopolitan world. Understanding the new world requires a cosmopolitan turn in the methodology of social sciences, a transition that compels breaking with methodological nationalism (Beck, 2006; Beck and Sznaider, 2006).

## The Social Science of Risk Society: Cosmopolitanism, Cosmopolitan Outlook, and Methodological Cosmopolitanism

The theory of risk society has provided a new ground for sociology to revisit many of its assumptions in relation to the new global terrain and thus make many current trends and developments comprehensible. Risk society is reflected as a new phase of modernity in which conflicts over what Beck refers to as the “bads” of modern industrial societies have replaced the “goods” that were formerly desired. This is while many of the so-called “goods” remain and are still considered desirable. Now, they involve the threatening and immeasurable side-effects and what are considered as “externalities.” Beck tries to demonstrate a special situation in which the attempt of modern man to comprehend as well as control over the world, has far-reaching consequences and threats for modern society. These consequences and side-effects and their relevant cultural aspects have given rise to a new transformation in modernity and have almost paved the way for a new reconfiguration of modern society. This transition in the ontological aspects requires a shift in the epistemological premises of modern social sciences. By changing the social ground, boundaries, behavioral and intellectual patterns, as well as emerging the new social entities and forces, the existing theories, and apparatuses of conceptualization and categorization of sociology must also transform, a transformation that could make sense of the various aspects of the world risk society.

By distinguishing cosmopolitanism from other notions like universalism, globalism, transnationalism, and internationalism, Beck strives to go beyond the conventional conceptions of the notion. For him, “cosmopolitanism is an ideal and a reality—and a synthesis of both—of universalism that retains a particularistic dimension, of globality that includes nationalism, and of transnationalism which does not exclude a plurality of local ethnicities and of cultures” (Beck, 2006, p. 145; Beck, 2016c, p. 29; Beck and Sznaider, 2006, p. 399–400; Blank, 2014, p. 66). In this definition, on one side, the question of “what is cosmopolitan?” takes precedence over “what it should be?” (Beck, 2006, p. 44; Beck and Sznaider, 2006, p. 384), in another side, cosmopolitanism, rather than being antithesis, is the synthesis of various categories, likes locality, nationality, globality, etc. (Beck, 2006, p. 57–58). In this case, many of the dominant dualities in the social sciences, such as local/global, national/international, and etc. are suspended (Beck, 2006, p. 383).

For Ulrich Beck, the cosmopolitan outlook offers the sociology for the second modernity and risk society. Cosmopolitan sociology is been constructed based on a transition “from a nation-state definition of society and politics to a cosmopolitan outlook” (Beck, 2016a). The cosmopolitanization of reality becomes the foremost subject for the social inquiry, a concern that will bring special conceptual, methodological, empirical and normative implications for the social sciences (Beck and Sznaider, 2006). Beck seeks to demonstrate the necessity of cosmopolitan sociology based on a gap, a distinction, a lag, and an asymmetry between what really exists in the social world and what we take for granted or scrutinize in our theoretical, conceptual, and even ethical approaches. The first step in facing this issue is to identify the really-existing process of cosmopolitanization of the world. For him, “Cosmopolitanism is equated with reflexive cosmopolitanism”, a reflexivity that itself, on the one hand, emerges from this gap and on the other hand paves the way for the creation of a new space on a global scale in which even old cosmopolitan ideals could and should be transformed and re-configured in respect to the really-existing, concrete social realities (Beck, 2006, p. 386).

Cosmopolitanism is a historical moment that is crystallized in the specific conditions of the current world. By distinguishing between “cosmopolitanism as a set of normative principles and really existing cosmopolitanization”, Beck tries to reject the claim that cosmopolitanism is “a conscious, deliberate and voluntary select, and all too often the choice of an elite”. Rather, the term “cosmopolitanization” was adopted to underline that “the emerging cosmopolitan of reality is also, and even primarily, a function of coerced choices or a side-effect of unconscious decisions” (Beck, 2006, p. 18–19; Beck, 2016c, p. 19–20; Beck and Sznaider, 2006, p. 387–388). In other words, the perspectives on cosmopolitanism have not paid enough attention to the fact that, in addition to the intended, there is an unintended and lived cosmopolitanism, which is becoming increasingly important. The growing transnational interconnections among different social actors particularly implies that the really-existing “cosmopolitanization” takes place as “unintended and unseen side-effects of actions which are not intended as ‘cosmopolitan’ in the normative sense” (Beck, 2006, p. 7, 111; Beck and Sznaider, 2006, p. 387).

Beck considers the aforementioned conditions as the cosmopolitan conditions, a latent cosmopolitanism that is been realizing at a historic moment, a moment that he calls the cosmopolitan moment (Beck, 2007a). What is happening here is “a *global awareness, a* self-conscious political affirmation, its reflection and recognition, a reflexivity that makes the ‘cosmopolitan outlook’ one of the key concepts and topics of the reflexive second modernity” (Beck, 2006, p. 21; Beck and Sznaider, 2006, p. 390). But under what conditions does this moment realize and “What are the characteristics of the cosmopolitan moment”? Beck deals with these questions by referring to his theory of *World Risk Society* (Beck, 1999b). The current world risk society is exposed to various but interdependence crises and risks, the most important of which for Beck are ecological, economic, moral and terrorist interdependency crises. What these crises have in common is that “they cannot be construed as external environmental crises but must be conceived as culturally manufactured actions, effects, insecurities and uncertainties.” In this respect, global risks can promote “global normative consciousness, generate global publics and advance a cosmopolitan outlook” (Beck, 2006, p. 22–24; Beck and Sznaider, 2006, p. 391). In world risk society the question regarding the causes and agencies of global risks and threats trigger new struggles, which in turn sharpen an “*institutionalized* cosmopolitanism” in conflicts over definitions, policies and jurisdictions (Beck, 2006; Beck and Sznaider, 2006, p. 391–392). This is the moment of cosmopolitan revelation that will accompany with various ontological, epistemological and methodological consequences for society and politics as well as social sciences. Thus, understanding the cosmopolitan moment requires to adopt a cosmopolitan outlook. In general, Beck distinguishes five interrelated constitutive principles of the cosmopolitan outlook as follows (Beck, 2006, p. 7):

First, the principle of *the experience of crisis in world society*: the recognition of interconnectedness and the consequent “civilizational community of fate” triggered by global dangers and crises, which transcends internal and external, us and them, national and international boundaries;

Second, the principle of *recognition of cosmopolitan differences* and the subsequent *cosmopolitan conflict character*, and the (restricted) concerning toward cultural and identity distinctions;

Third, the principle of *cosmopolitan empathy* and of *perspective-taking* and the virtual interchangeability of situations (as both an opportunity and a threat);

Fourth, the principle of the *impossibility of living in a world society* without boundaries and the resulting compulsion to redraw old boundaries and reconstruct previous barriers;

Fifth, *the mélange principle*: Local, national, ethnic, religious, and cosmopolitan cultures and traditions interpenetrate, interact, and intermingle—cosmopolitanism without provincialism is hollow, and provincialism without cosmopolitanism is blind.

The translation of cosmopolitan outlook for social sciences and theory is containing an especial focus on the “analytical-empirical cosmopolitanism” which concurrently demarcates itself from “normative-political cosmopolitanism,” but at the same time presupposes it. This distinction advances an objective approach to concrete experience and the epistemological aspect of world risk society in the social sciences. This is the very moment that the relation between the practical and theoretical categories of the cosmopolitan outlook or the critique of methodological nationalism, on the one side, and the normative cosmopolitan and the politics of cosmopolitan on the other side, is being called into question (Beck, 2006, 2016a; Beck and Sznaider, 2006).

Reaching the analytical-empirical cosmopolitanism and grasping the cosmopolitanization of reality as the main concern of cosmopolitan sociology, requires the critique of methodological nationalism and then a transition to methodological cosmopolitanism (Beck, 1999b, 2006, 2007a, 2009, 2016a; Beck and Sznaider, 2006). Methodological nationalism For Ulrich Beck,

…* takes the following premises for granted: it equates societies with nation-state societies and sees states and their governments as the primary focus of social-scientific analysis. Methodological nationalism assumes that the nation, state and society are the “natural” social and political forms of the modern world. It assumes that humanity is naturally divided into a limited number of nations, which organize themselves internally as nation-states and externally set boundaries to distinguish themselves from other nation-states. And it goes further: this outer delimitation as well as the competition between nation-states, represent the most fundamental category of political organization (Beck*, *2006**, p. 24; Beck and Sznaider*, *2006**, p. 383)*.

He also added that,

*In effect, the social science stance is rooted in the concept of the nation-state. It is a nation-state outlook on society and politics, law, justice and history, that governs the sociological imagination. And it is exactly this methodological nationalism that prevents the social science from getting at the heart of the dynamics of modernization and globalization, both past and present: the unintended result of the radicalization of modernity is a disempowerment of Western states, in sharp contrast to their empowerment before and during the nineteenth-century wave of globalization (Beck*, *2007b**, p. 287)*.

In methodological nationalism, categories in theory, take precedence over categories in practice. The slogan of cosmopolitan turn is “Back to the things themselves! Away from pure theories for their own sake!…” (Beck, 2006, p. 75; Beck, 2016b, p. 463). Criticism of methodological nationalism never implies the assumption of the end of the nation-state, but rather believes that the role and position of these political units in practice has changed profoundly, and that this transformation must be taken into account in social sciences. More precisely, it can be revealed that “the national organization as a structuring principle of societal and political action can no longer serve as the orienting reference point for the social scientific observer” (Beck and Sznaider, 2006, p. 384). Social sciences as a prisoner of the national view and the nation-state, are producing dead categories that no longer have the efficiency to grasp global social terrain. Beck calls these categories the “Zombie Categories”, social, dead categories that are not adequate anymore to grasp social realities, categories, such as class, family, gender and nation, that have been constructed in the world of nation-states and nationalisms (Beck, 2001, 2002; Gross, 2016). The national view confines not only what we can imagine and desire, but, more prominently, what we know and what we think the reality is. Our data, statistics and categories are all subject to the national perspective. And it is the main aim of the cosmopolitan outlook to liberate the social sciences from the control of the national outlook. The most basic and prominent categories in social sciences, such as individual, family, gender, religion, class, poverty, inequality, state, politics, democracy, law, etc. are all nationally defined. The de-mystification of the social sciences would compel us to part with nationalism and touch the contemporary world as it is: already cosmopolitan (Beck, 2006; Beck and Sznaider, 2006; Blank, 2014).

Cosmopolitan sociology is primarily concerned with the dismantling of the mutual interrelationships between the national outlook of politics and society, and the methodological nationalism of social sciences, an interrelation in which they acknowledge and reinforce each other in the definitions of reality. It also addresses numerous growing versions of de-bounded and transnational social and political entities, along with their respective epistemological and methodological implications. National entities and spaces have become de-bounded, and the national will lose its implication, just as the international will lost its meaning (Beck, 2006). New entities and realities are emerging: a new temporal and spatial figuration, as well as a new social and political configuration, which must be explored from theoretical and empirical viewpoints. This necessitates a rethinking of the core ideas of “modern society” (Beck and Sznaider, 2006, p. 386). By giving priority to the empirical dimension, all existing social categories, which here are considered as zombie categories, have to be freed from the chains of methodological nationalism and re-categorize within the framework of methodological cosmopolitanism and new cosmopolitan social sciences.

Beck put his emphasis on three issues in methodological cosmopolitanism: alternative, non-national unit of research; cosmopolitan understanding; and cosmopolitanism between universalism and relativism (Beck and Sznaider, 2006; Beck, 2007a). Liberating from the cage of methodological nationalism will be accompanied by the significance of de-territorialized, global, de-bounded and transnational entities. Here the unit of research is extracted from the within of the concrete world and is spatially and temporally meaningful in respect to the specific reality, the reality which is constructed within a cosmopolitan world in never-ending relations with other global realities (Beck and Sznaider, 2006, p. 394–397). Back claims that units of research in cosmopolitan sociology can be identified with respect to two distinctions: the first distinction is related to whether the units refer to processes of transnationalization or to transnational structures, and the second distinction might be related to the range and the location of cosmopolitization (Beck, 2016c, p. 25–26; Beck and Grande, 2010, p. 428).

Beck proposes three possibilities for prevailing the cosmopolitan units of research in social sciences, including the replacement of the national with transcontinental processes; the substitution of the national with new transnational structures and focusing on transnational networks and border zones; and finally, the embedment of the national in new transnational structures and processes (Beck, 2016c, p. 27–29; Beck and Grande, 2010, p. 428–432). As Beck and Grande put it, “the transnational spaces, processes and structures which constitute the units of analysis in a cosmopolitan methodology can construct the units based on historical; functional; social; or institutional criteria”. Politics of remembering or transnational spaces of remembrance can be instances for transnational, historical units. Zeroing in on the transnational policy regimes would refer to functional-based units of analysis. By taking social practices and actions, in particular social struggles and conflict structures, as a central point of cosmopolitan analysis, social aspect of cosmopolitanism can be addressed. And finally, transnational units of analysis could be recognized by novel forms of transnational institution through their construction (Beck and Grande, 2010, p. 429–432).

Grasping various relations between different social entities in the world risk society is very crucial for the cosmopolitan understanding. According to Beck, methodological cosmopolitanism must conceptualize and categorize the relational patterns of the “transnational”, “global–local”, “global–national”, “national–global” or “global–global” with local, national, transnational and global focuses. By concentrating on the cosmopolitan understanding, Beck strives to privilege a kind of cosmopolitan hermeneutics (Beck, 2006; Beck and Sznaider, 2006, p. 397–398). It is a multi-perspective and boundary-transcending approach toward the complex and relational global realities. In this respect, realistic cosmopolitanism is at the forefront of idealistic cosmopolitanism and while recognizing and being sensitive to certain differences and particularities across cultures, “presupposes a universalistic minimum including a number of substantive principles and norms” (Beck and Grande, 2010, p. 417–419; Beck and Sznaider, 2006, p. 399–400).

## World Risk Society as Unit of Reference for Bourdieusian Theory of Practice and Fields

As mentioned above, Ulrich Beck strives to conceptualize a novel world with a new conceptual apparatus. This world, which has become fluid and uncertain due to the consequences of the collective actions of modern man and their self-reflection as well as the expansion of globalization, has acquired cosmopolitan and transnational implications. But is the cosmopolitanization of social realities itself a novel construction within a new ground? From an ontological point of view, put differently, has been new social being emerged? That is, we transit from one ontological state to another ontological state? According to Beck, cosmopolitaness, contrary to the dominant ontology in the social sciences, which were based on the logic of either/or, implies the logic of and/both. On this basis, all categories and realities are cosmopolitan if they are not constructed solely on a specific reality or exclusively on a particular category. What will be the consequence of this general ontological proposition for social theory as well as social sciences? Are we facing the end of social ontology? But as Beck indicates in his cosmopolitan outlook, cosmopolitanism is determined and crystallized based on the specific conditions and certain moments. Emphasizing the cosmopolitan moment means prioritizing a certain time, space, and reality, as well as actors and a set of special actions, a moment to which Beck's cosmopolitan apparatus is silent, especially in dealing with the empirical analysis of concrete cosmopolitan realities. Since proposing a fixed and sound theoretical apparatus, by sacrificing social reality, ultimately falls into the trap of essentialism, Beck himself seems reluctant to offer a determined categorical apparatus as well as empirical analysis. However, his theoretical premises and promises in the theory of risk society and methodological cosmopolitanism do not fit with his passive confrontation with concrete realities. This problem is clearly evident in the efforts of Beck et al. (2013) in examining the formation of cosmopolitan communities, that is, cosmopolitan communities of climate risk. Due to the incompleteness of his ontology as well as the methodological defects of cosmopolitan theory in facing concrete realities, he has to employ Benedict Anderson's idea of imagined communities to take into account these communities, an exercise which is itself highly controversial. However, these problems have been overcome to a high degree in the former empirical studies (see Blok, 2016; Zhang, 2018).

If the main outcome of Beck's conceptual apparatus is getting step into the transnational as well as uncertain and ambivalence world, based on the Pries' triple units in the study of social phenomena in a global ground (Pries, 2007), it can be clearly seen that our unit of reference has transformed, but in the other two units, namely units of analysis and measurement, we are still experiencing serious epistemological and methodological problems. If the cosmopolitan moment is the simultaneous moment of combining and transcending different types of social realities, then is this dynamic in different temporal and spatial conditions, social contexts and especially according to the intensity and extent of various realities, such as ethnic, local, national, regional, etc., follows a single and universal logic? Are the consequences of global warming for a country in the global south dominated by a totalitarian state the same as those for a northern metropolis? Although the risk is neutral with respect to ethnicity, gender, class, level of development, and other social categories and distinctions, we do not experience the same reactions from different social groups at different times and places. A unique example, in this case, is the Covid-19 pandemic. This phenomenon, which was local at the time of its occurrence, immediately became a transnational phenomenon and rapidly engulfed all societies around the world. No country was spared in this crisis, and on the other hand, they could not deal with it in isolation. Each society gave specific reactions according to its national and local characteristics and coordinates, multiple reactions that were not constant even in time. These reactions were the source of the formation of various social configurations, especially institutional configurations (Mosleh and Jong, 2021). Could we call all these configurations cosmopolitan? If we highlight the very logic of the cosmopolitan moment, we find that societies are experiencing different types of cosmopolitan configurations. The configurations are contingent on social contexts and, according to Beck's theoretical premise, could only be understood by empirical analysis and only within the boundaries of those configurations.

Many of the categories and notions proposed by Ulrich Beck are contingent, indeterminate, and uneven. This means that they are determined in certain realms of society and at a specific period. In other words, in some social sectors, risks can be the source of wide-ranging transformations; or individualization and self-reflection in some areas are more pronounced than in others; or cosmopolitanization has affected some cultural and social spaces more than others. Hence Beck's social theory could be called the general knowledge of the indeterminate or the uncomplete social ontology of the indeterminate. Whereas, his theory is indifferent to the social distinctions and categories in different societies and is not able to make sense of the distinctions neither conceptually nor empirically. The issue can be tracible in his universal and positive statements about the three main axes of methodological cosmopolitanism. On the one hand, he considers cosmopolitanism to be a really-existing reality that can be understood by criticizing methodological nationalism and turning to methodological cosmopolitanism as well as deconstructing the zombie categories. But from a prior position and positively, he attempts to impose a special conception of the transnational unit as the legitimate unit of cosmopolitan analysis. In addition, in cosmopolitan understanding, he addresses a limited and unilateral hermeneutical understanding as well as relationships, and finally, he positively attributes a scale for cosmopolitanism between universalism and particularism. This predicament has also led to the dominance of a kind of Eurocentrism in the idea of the second modernity and cosmopolitanism (Mythen and Walklate, 2016; Zhang, 2018). According to Beck' premises, in general, the cosmopolitan empirical-analytical approach takes precedence over other perspectives. This means that categories, units of analysis, and their scale must be derived a posteriori and empirically from social realities. Any understanding of cosmopolitan reality is contingent on a special social reality. Highlighting cosmopolitan conditions and moments means that cosmopolitanism is essentially a relational and contingent reality to certain conditions as well as other social realities, a relationality that must also be dominated in cosmopolitan understanding. These predicaments in Beck's theory of risk society and cosmopolitanism have led many researchers to select parts of his theories and notions in relation to their research aims (Mythen and Walklate, 2016; Mythen, 2018).

The incompleteness of Ulrich Beck's social theory must be complemented by a theoretical apparatus in which the agency, positionality, relationality, and contingency of social realities are significant. On the one hand, concerning the similarities of some of Beck's premises and promises with Pierre Bourdieu's theory of practice and fields, and on the other hand, given the relational and non-essentialist nature of Pierre Bourdieu's approach, the present article argues that the theory of global risk society can be promoted by putting it as the unit of reference for Bourdieusian notions and theories, a replacement that itself will be the basis for a serious critique of the theory of risk society and cosmopolitanism. Bourdieu's multiplied relational approach and its focus on constructing everyday realities—a vision from below contrary to Beck's elitism or what Vara calls “a vision from above” (Vara, 2015)—at the empirical level could resolve many of the predicaments mentioned in Beck's social theory. In his latest book, *The Metamorphosis of the World* (Beck, 2016c), Ulrich Beck explicitly considers Bourdieu's social theory to be an instance of a social theory in which methodological nationalism, as well as metaphysics of reproduction, have dominated. According to Beck, Bourdieu's fields are configuring within the framework of nation-states, and in addition, he considers his theory of practice, especially Habitus, as a manifestation of Bourdieu's submission to the status quo and the lack of attention to profound changes in the social order. Beck's critiques of Bourdieu's social theory may are true to some extent, but basic philosophical premises of Bourdieu's theory, and especially new re-visiting of Bourdieu (Lamont, 2000; Lamont and Molnár, 2002; Wimmer, 2002; Brubaker, 2004; Pachucki et al., 2007; Go, 2008, 2013; Go and Krause, 2016), have been able to overcome these problems.

In this regard, several key propositions could be extracted from Beck's theories and notions which are elaborated in this article. Modernity and industrial society have entered into a new state, a situation in which the threats arising from human activities have become the source of new transformations and crises. Contrary to other social forces and phenomena, the risk is neutral to time, space, ethnicity, gender, class, level of development, and other social categories and distinctions. “We are somehow on an equal basis in the face of the impending catastrophe: new ties, stronger and all encompassing, emancipate us from old ties. There are no masters and slaves when the whole house might go on fire”(Vara, 2015, p. 101). Hence, risk has been pervasive and affected different social strata, institutions and structures and challenges their authority. In this uncertain world, a kind of unbridled individualization prevails and actors must choose and face the consequences of their choices on their own and without any institutional support. This gives rise to a new kind of self-reflection and subjectivity. This trend has expanded thanks to globalization and by deconstructing many social realities, has paved the way for a kind of global transformation in different societies. In this context, the confrontation with the side effects of the first modernity provides the ground for the formation of cosmopolitan realities and awareness. From a non-Eurocentric approach, the consequence of this process can be considered in Chang Kyung-Sup's expression as compressed modernity or internalized reflexive cosmopolitization (Kyung-Sup, 2010). In this reading, Beck's reflexive modernity points out to a civilizational condition in which “economic, political, social and/or cultural transformations take place in an extremely condensed manner in respect to both time and space, and in which the dynamic coexistence of mutually disparate historical and social elements leads to the construction and reconstruction of a highly complex and fluid social system” (Kyung-Sup, 2010, p. 446). Different societies co-opt cosmopolitanized risks, which reflectively encompasses all aspects of social life. As a result, societies with diverse characteristics are integrated into one another, resulting in compressed modernity which becomes a universal trait of contemporary societies. Ontologically, therefore, we are confronted with the absence of a determined and lasting reality rather than a new reality. In this setting, side effects and risk become the central element in shaping new social realities. Individualization prioritizes agency over structures and makes actions and practices the organizing elements. The indeterminacy and contingency of social realities cause the cognitive categories to become temporarily and spatially conditional to the empirical reality and their forming conditions. The actor is thought to be rational and free to choose. Under the new global context, new social realities are constructed by deconstructing, combining and reconstructing existing realities, realities that are fundamentally transnational.

Risks and crises can directly or indirectly give rise to social realities, social categories, identity patterns, cultural forms, habitats, practices and actions, distinctions, fields, new capitals, capital exchange rates, and rules of the game. Bourdieu and Bourdieusian approaches, in the light of the relational perspective, examine these notions as well as their process of constructions and transformations in different social contexts. Thus, from a relational standpoint, we can name the cosmopolitan social realities, categories, identity patterns, cultural forms, habitats, actions, distinctions, fields, new capitals, capital exchange rates, and rules of the game. In the Bourdieu approach, the emphasis on the contingency makes the condition of possibility of social realities the primary concern of social inquiry. Based on the Beck's own words, thus, the cosmopolitan moment, which is the accumulation and expansion of risk on a large scale, is the decisive moment or the constructive condition for emerging cosmopolitan realities. The moment is accompanied by, “the increasing fragmentation of the social (the dis-embedding effect of risk) and the potential instrumentality of a heightened awareness of global interrelatedness (the re-embedding effect of risk)” (Zhang, 2018, p. 71). Zhang argues that what is key in this process is “how reflexive dialogue with global experience internally transforms social agencies” (Zhang, 2018). Whereas, where risks can bring about wide-ranging changes, individualization and self-reflection are deeply embedded, and the cosmopolitan conditions are broadly institutionalized, these realities could emerge. Actors interact with each other in creating social realities under the unequal distribution of valuable resources, the distribution of which finds symbolic expression in cultural forms and practices.

According to Bourdieu, “The object of social science is a reality that encompasses all the individual and collective struggles aimed at conserving or transforming reality, in particular those that seek to impose the legitimate definition of reality, whose specifically symbolic efficacy can help to conserve or subvert the established order, that is to say, reality” (Bourdieu, 1990, p. 141). Now realities are constructing, deconstructing and reconstructing in the new social ground, namely in world risk society and through the reflexive and cosmopolitan order of realities. Based on the different contextual distribution of valued resources, and in the light of the role-playing of risks, social actors through encountering with new social world (position) in the different “social fields” (structural basement), internalize its values, categorizations and distinctions (disposition), and then acting spontaneously and intentionality by these categorizations and distinctions (practice), reproduce or deconstruct the social structures through values and meanings they have internalized (Bourdieu, 1990; Jenkins, 1992; Todd, 2005). But there is no direct relation in this process, it would be relative to the temporal and spatial context, social field, actors' accessibility to resources and groups. On the one hand, risk can be a forcing factor that is the source of either the creation of new realities, awareness and social relations or the transformation of existing realities, and on the other hand, risk itself can be the output—side effect—that arises from new realities and relationships. The effect of risks and threats on the actors' position leads to constructing various perceptions, the perceptions that would affect the dispositions and then practices of the actors. The process of self-reflection takes place in exactly the very process. It is from this point on that disputes and negotiations over the quiddity and meaning of risk among different actors begin.

Bourdieu assumes an unequal allocation of cultural, political and economic resources, which he considers as social structure (positions) (Bourdieu, 1990). Individuals as encountered the objective world, internalize their positions in this structure by steadily developing a habitus fitted to these positions. The habitus is a system of “pre-dispositions” that establish actions, perceptions and interpretations regarding related social positions (Bourdieu, 1990; Bourdieu and Wacquant, 1992). The outcome of this process is construction of “a collective of strategies for action and cognitive patterns” (Wimmer, 2002, p. 27). In a strong Bourdieusian sense, Andreas Wimmer draws our attention to the empirical representation of habitus, which he named “Scheme”. “Schemes are models of simplified worlds, he argues that ordered as interrelated networks of meaning and conception and they are spontaneously selected and activated in everyday perception, consciousness, thinking and action” (Wimmer, 2002, p. 27–28). People internalized these schemes of cognition within their own lifeworld through socialization processes. In the global risk society, habitus must be understood as “being formed on the basis of a universal human competence—which is not determined by specific culture—, the competence of assessing pros and cons in given situations in light of one's own interests” in terms of their perceptions of risk and the role of other actors in controlling the risk's effects. The perception and evaluation of “*what* one's own interests are” is actually relied on primary regulations to cultural backgrounds and one's own social position. These are, eventually, merged in the individual's habitus (Wimmer, 2002). In respect to the transnational character of risk, people could spontaneously evaluate their own positions in relation to other actors and then based on their evaluations design different strategies for their practices to reduces the effects of different risks. Here Cosmopolitanism can be an outcome of this process among other possibilities. This means that actors' assessments and their shared conceptions of the quiddity, origins, and consequences of risk lead them to engage in cosmopolitan relationships and cooperation.

When virtually “habitual schemes” are customized to the distinct positions within a society, they construct different categorizations, classifications, evaluations and world-views. In this ground, they produce elements and categories which all actors who are involved can identify as “congruent” to their corresponding interests (Wimmer, 2002, p. 28). Wimmer holds to the idea that this negotiation process eventually leads to a sphere that he called “cultural compromise”. With considering the notions of negotiation and consent, he defines cultural compromise “as consensus over the validity of collective norms, values, categorizations and patterns of interpretation that persists beyond the open and never-ending process of its construction” (Wimmer, 2002, p. 29). In everyday life and interactions, the actors negotiate: how a position ought to be defined, who should play what role, which plans for action should be chased, and which norms and values are relevant in the certain position. So, when people concur around some interests, then any binding rules for collective making-meaning over realities will develop (Bourdieu and Wacquant, 1992; Wimmer, 2002, p. 28–33). Therefore, a compromise on the quiddity of risk and its relevant effects will appear when all social actors, based on their interests, in relation to each other in a commutative field, come to a consensus around its respective social categories and classifications and then they are trying to make them legitimate and valid. The outcome of this process would be different types and orders of social categories in relation to risks, and their relevant meaning formations, like patterns of identity, ethnicity, gender, etc. into different layers of risk society.

The process of cultural compromise around the meaning and effects of risk paves the way for drawing boundaries between different social actors. The point is that a complete cultural compromise would be constructed if all social actors in relation to each other in a social field can formulate dimensions of their interests in “a shared symbolic language” (Bourdieu, 1990, 1991; Wimmer, 2002). Conclusively, an outcome of this compromise would be “certain cultural and also social markers” which are singled out in order to expose and support the distinction between insiders and outsiders—between those who are in the same compromise and those who are staying on the margins. Social closure could result in the formation of different social groupness like classes, gender-defined groups, subcultures, or ethnic groups, nations, transnational diasporas and cosmopolitan groups. The boundaries between “us” and “them” are frequently marked by distinguishing shapes of everyday cultural and social practice (Lamont and Molnár, 2002; Wimmer, 2002; Brubaker, 2004; Pachucki et al., 2007). This is the social reality moment, the reality that can be national, transnational and cosmopolitan according to the habitual schemes as well as the order of cultural compromises and categories, and social boundaries. The very logic of the formation of social realities can be traced in the process of establishing many cosmopolitan risk communities. In this regard, Zhang (2018), for example, in her empirical study of China's good food movement as a cosmopolitan risk community, indicates how the Chinese middle class, in response to risks associated with China's food industrialization as well as the media coverage of this problem, were able to establish a cosmopolitan risk community with new relationships, beyond the previous ones. According to Zhang, the cosmopolitan moment or the condition of possibility of this non-hierarchical cosmopolitan movement was not the basis of the density and scale of risk, but “the ‘intimacy’ of risk that impelled the coming together of a cosmopolitan community” (Zhang, 2018, p. 76). By understanding the types and distinction of “traditional” and “new” risks associated with contemporary food consumption, and by adopting and hybridizing similar relations and elements from the same currents in other countries, they could reorganize social relations within the food system and establish “community resilience to food-borne risks” (Zhang, 2018, p. 75). Through the process, new risk perceptions, habitus, solidarity, cultural compromise, and social closure have been constructed.

On the other side, fields are the structures in which social realities emerge and endure. In the risk society, risks can affect either the existing fields and reconfigure them, or configure new fields, or deconstruct other fields. For Bourdieu, fields are realms of struggle in which actors compete for a variety of valued resources, that is, different species of “capital” that are potentially convertible to each other in the various exchange rate (Bourdieu, 1984, 1990, 1991; Bourdieu and Wacquant, 1992). Fields are made up of two dimensions that are linked but conceptually distinct: “first, the objective configuration of actor-positions and second, the subjective conceptions and meanings that direct actors in the conflict”, i.e., the “rules of the game” and certain forms of cultural or symbolic capital (Bourdieu and Wacquant, 1992, p. 97; Go, 2008, p. 206). Individuals or corporations may be considered as the actors of the field; the “capitals” desired by any given actor in the field may be diverse (e.g., economic, political, or symbolic capital); and the “rules of the game” may differ between fields or across time (Go, 2008, p. 207). Some capitals and their accumulation, as well as special exchange rates, could possess a direct outcome for reducing the impact of risks. Here we can also mention cosmopolitan fields, that is, fields that have arisen as a direct result of global risks such as global warming, as well as fields that have been formed to deal with these threats. These fields are beyond national and even international fields and cover a wide range of human communities as they are widespread through time and space (Go, 2008; Go and Krause, 2016). In this case, the risk-based fields, as well as the cosmopolitan fields are contingent fields, that are configured concerning other fields and could be of their own distinct rules of the game.

What is really important here is, on one side, the capitalization of risk, and one the other side, the different perceptions of risk for different people in different places which constructs variety of social fields. In a distinct study in this respect, Vara (2015) take into account different effects of the environmental crisis on lithium mines in Chile, Bolivia, and Argentina, and shows how capitalized risk has given rise to the formation of fields as well as various social movements in Latin America. Encountering and reflecting the potential risks as a result of the crisis in this site has led to the development of a kind of horizon of equality and redefining economic relations in these countries, as well as their global role by their officials. Here, for these states, active participation in the issue of lithium mining is tantamount to cosmopolitan action and symbolic capital for greater autonomy and equality. On the other hand, according to Vara, this crisis, as well as the reaction of governments at the national level, has been exacerbated by the reaction of various classes from the simple workers to the intellectuals, and as a result new fields, symbolic capitals, movements, organizations and protests have been formed.

## Conclusion

Rather than being a risk theory in modern society, the theory of Risk Society is a social theory of the new social world. Although the process of modernization had brought tangible achievements and progress to modern man, as Ulrich Beck mentions, in parallel with these developments, modern society was facing unprecedented crises and threats. In contrast to the first modernity, the risks and side effects of human activities, which now grieve many aspects of human life, are the prevailing and determining element of the new world. These risks and threats have imposed a distinctive character on this world and hence, have led to a profound transformation in social forces and relations. The result of this process is the supremacy of a sense of distrust, anxiety and uncertainty over modern society. This is the emergence of a situation that Paul Virilio (2009) calls a catastrophic society. According to him, progress and catastrophe, or in the words of Ulrich Beck, side effects are two sides of the same coin. Now, “the project of modernity has faced its limit in the light speed colonization of terrestrial time and space by technology and media, and has now started to contract back toward a singularity of infinite density that is uninhabitable for embodied humans and only really liveable as virtual or spectral space” (Featherstone, 2010). Social phenomena and events under the empire of speed can immediately take on a destructive character and pervade vast times and spaces (Armitacge, 1999). In this respect, understanding and reflexing on the critical situation, that has become more and more evident in the relationship between humans and the environment, finds a special signification. In the light of a high-speed society and a world of flows, knowledge and all kinds of theoretical apparatuses have lost their previous implication, and new cognitive configurations are thus essential in taking into account the individual as well as social aspects of current catastrophic society (Virilio, 2008).

What Beck emphasizes is that the social sciences and social theory need a fundamental overhaul to understand this new society and the corresponding transformations that have developed by globalization. Contrary to the historical trend of social theory, global transformations have reached such a level and depth that it is no longer possible to modify theories solely on the assumptions of a static society specific to the first modernity, namely the world of nation-states. Here, social theory and its empirical implications must evolve in such a way as to be able to understand the nature of indeterminacy, fluidity, reflection, as well as the universality of social reality and trends. The epistemological and conceptual apparatus proposed by Beck for this new world is cosmopolitan sociology. In this new conceptual apparatus, the practical categories, as well as the immanent nature of phenomena, are prioritized. Here, unlike conventional sociology, where the main focus was on positive outcomes such as growth and development, the main emphasis puts on the bads and side effects of modern human actions. The unbridled individualization and decline of the institutions' authority in the first modernity, and along with the heightening of new self-reflection, lead to the formation of a new subjectivity and its prevailing over social structures. This process underlies the cosmopolitanization of realities, which Beck believes could only be understood in the light of the cosmopolitan outlook.

In sum, Ulrich Beck's theoretical reflections comprise, on the one hand, an attempt to prove a new world along with new social ontology, and on the other hand, an emphasis on the need for a fundamental epistemological shift in the conventional social sciences. As this article has shown, this significant shift in the conceptual apparatus proposed by Ulrich Beck is not fully compatible with his social ontology, and this has led to the incompleteness of Beck's social theory and ontology in understanding the world risk society. By juxtaposing the risk society—as a unit of reference—and Pierre Bourdieu's theory of action and fields, in dealing with this issue, an attempt was made to overcome some of Beck's epistemological problems. Through this juxtaposition, on the one hand, the contingentness of the occurrence of many of Beck's notions and categories in the concrete world is effectively illustrated, and on the other hand, the really-existing divisions of social realities could also be considered.

## Author Contributions

The author confirms being the sole contributor of this work and has approved it for publication.

## Funding

This article processing charge was funded by the Deutsche Forschungsgemeinschaft (DFG, German Research Foundation – 491192747) and the Open Access Publication Fund of Humboldt-Universität zu Berlin.

## Conflict of Interest

The author declares that the research was conducted in the absence of any commercial or financial relationships that could be construed as a potential conflict of interest.

## Publisher's Note

All claims expressed in this article are solely those of the authors and do not necessarily represent those of their affiliated organizations, or those of the publisher, the editors and the reviewers. Any product that may be evaluated in this article, or claim that may be made by its manufacturer, is not guaranteed or endorsed by the publisher.
